# Adenosine-Metabolizing Enzymes, Adenosine Kinase and Adenosine Deaminase, in Cancer

**DOI:** 10.3390/biom12030418

**Published:** 2022-03-08

**Authors:** Galina Zhulai, Eugenia Oleinik, Mikhail Shibaev, Kirill Ignatev

**Affiliations:** 1 Institute of Biology, Karelian Research Centre, Russian Academy of Sciences, 185910 Petrozavodsk, Russia; eokimm@mail.ru; 2 Endoscopic Department, Baranov Republican Hospital, 185019 Petrozavodsk, Russia; mshib@karelia.ru; 3 Surgical Department no. 2, Republican Oncological Dispensary, 185002 Petrozavodsk, Russia; kirillignatev@bk.ru

**Keywords:** adenosine, ADK-L, ADK-S, ADA, cancer therapy, tumor microenvironment

## Abstract

The immunosuppressive effect of adenosine in the microenvironment of a tumor is well established. Presently, researchers are developing approaches in immune therapy that target inhibition of adenosine or its signaling such as CD39 or CD73 inhibiting antibodies or adenosine A2A receptor antagonists. However, numerous enzymatic pathways that control ATP-adenosine balance, as well as understudied intracellular adenosine regulation, can prevent successful immunotherapy. This review contains the latest data on two adenosine-lowering enzymes: adenosine kinase (ADK) and adenosine deaminase (ADA). ADK deletes adenosine by its phosphorylation into 5′-adenosine monophosphate. Recent studies have revealed an association between a long nuclear ADK isoform and an increase in global DNA methylation, which explains epigenetic receptor-independent role of adenosine. ADA regulates the level of adenosine by converting it to inosine. The changes in the activity of ADA are detected in patients with various cancer types. The article focuses on the biological significance of these enzymes and their roles in the development of cancer. Perspectives of future studies on these enzymes in therapy for cancer are discussed.

## 1. Introduction

Cancer therapy based on the modulation of immune processes, such as immune checkpoint blockade and adoptive cell therapy, has achieved significant results. However, this approach does not work for most patients [1]. Therefore, the study of complex mechanisms of interaction between the tumor and the immune system and the search for new targets for therapy remains relevant.

Currently, the role of metabolism of purine nucleotides in the development of the tumor is well established. In normal conditions, adenosine is present in the human body in minor amounts (around 1 µM) and participates in various physiological processes including sleep, angiogenesis, and immune regulation [2,3]. Hypoxia, ischemia, and inflammation increase the levels of extracellular adenosine (>100 µM) in the tumor microenvironment (TME). In terms of oncogenesis, the adenosine pathway is considered to be a barrier to successful immunotherapy, which makes it an important therapeutic target [4]. Adenosine binds with adenosine receptors associated with G-proteins on the cellular surface. Their activation leads to the induction (A2A and A2B receptors) or inhibition (receptors A1 and A3) of intracellular production of cyclic adenosine monophosphate (cAMP). Stimulation of these receptors modulates the functions of inflammatory and immune cells, leading to the formation of immune-suppressive conditions in the TME, angiogenesis, and metastasis [4].

**Figure 1:**
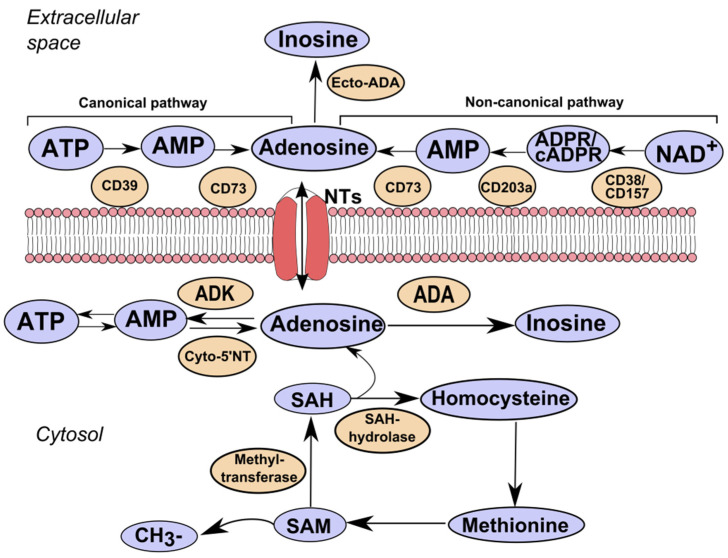
Adenosine metabolism. The canonical pathway of adenosine synthesis involves the hydrolysis of ATP to AMP by ecto-nucleoside triphosphate diphosphohydrolase (NTPDase1, CD39) and the hydrolysis of AMP by ecto-5′-nucleotidase (5′NT, CD73). The non-canonical pathway involves the use of NAD^+^ as a substrate by CD38 or CD157 to generate ADP-ribose (ADPR) directly or through its cyclic form (cADPR). ADPR is then processed to AMP by CD203a (Ectonucleotide Pyrophosphatase/Phosphodiesterase 1). Extracellular adenosine can bind to its receptors or be metabolized to inosine by ecto-adenosine deaminase (ecto-ADA). Adenosine is transported into and out of the cell by concentrative or equilibrative nucleoside transporters (NTs). Intracellular adenosine synthesis is controlled by the balance of the activity of enzymes: adenosine kinase (ADK), cytoplasmic 5′nucleotidase (Cyto-5′NT), adenosine deaminase (ADA). Adenosine is generated as an end product in the transmethylation reaction: transmethylation reactions include the transfer of methyl groups from S-adenosylmethionine (SAM) to a wide range of acceptors. The resulting product, S-adenosylhomocysteine (SAH), is then cleaved by SAH hydrolase (SAHH) into adenosine and homocysteine.

Extracellular over-expression of adenosine in the tumor tissue is primarily observed in extracellular adenosine triphosphate (ATP) released by the lesional tissue and immune cells [3]. While intracellular ATP is an important source of energy, extracellular ATP functions as a “signal of danger” directing phagocytes to the sites of inflammation and informing the immune system about pathogen-associated molecular patterns and tissue destruction. It occurs due to the activation of the inflammasome and the production of interleukin-1β and anti-inflammatory cytokines. Conversion of ATP into adenosine occurs due to the system of adenosine-producing enzymes, ecto-nucleoside triphosphate diphosphohydrolase (NTPDase1, CD39), and ecto-5′-nucleotidase (5′NT, CD73) that change inflammatory medium conditions to anti-inflammatory ones (Figure 1).

It was established that CD39, which hydrolyzes ATP into adenosine diphosphate (ADP)/adenosine monophosphate (AMP), and CD73, which mediates the subsequent breakdown of AMP into adenosine, plays a key role in the development of tumors [5]. Their expression and activity are elevated in the tumor tissue and blood and are often associated with clinical signs of the disease and unfavorable prognosis in some cancers [6,7,8,9,10,11]. During the past years, along with the canonical pathway of adenosine production by the ATP/CD39/CD73 axis, an alternative pathway for the formation of adenosine from extracellular nicotinamide adenine dinucleotide (NAD+) is discussed in cancer progression [12,13,14].

The levels of adenosine are regulated by adenosine-converting enzymes adenosine kinase (ADK) [15] and adenosine deaminase (ADA) [16]. ADK adds the residue of phosphoric acid to adenosine and converts it into AMP. ADA separates an amino group from adenosine with the formation of inosine. ADA Michaelis constant (Km) for adenosine is 25–150 µM (depends on the isoform), while ADK Km is approximately 1 µM. Since ADK affinity to adenosine is higher, ADK is considered to be the enzyme responsible for the regulation of the level of adenosine in physiological conditions [17]. Besides, the level of adenosine is controlled by bidirectional nucleoside transporters [3,18].

Currently, the therapeutic potential of inhibition of ectonucleotidase enzymes CD39 and CD73, that breaks down ATP to adenosine, and adenosine receptor A2A is established in preclinical studies and is being tested in phase I/II of clinical studies on oncologic patients [4]. These targets (CD39, CD73, A2AR) are actively studied. However, in the development of the adenosine-regulating approach, numerous enzymatic pathways that control the balance of adenosine-ATP, are understudied. Besides, intracellular regulation of adenosine is underestimated. Thus, the study of other components of adenosine metabolism and search for their associations with immune disorders in the conditions of tumor development are relevant. This review contains the latest data on the effect of adenosine on cells in the TME. It focuses on enzymes involved in the metabolism of adenosine-ADK and ADA and provides an overview of biological significance of ADK and ADA and their role in the development of tumor. Perspectives of further studies on these enzymes for the therapy of oncologic diseases are discussed.

## 2. The Effect of Adenosine on Cells in the Tumor Microenvironment

**Figure 2:**
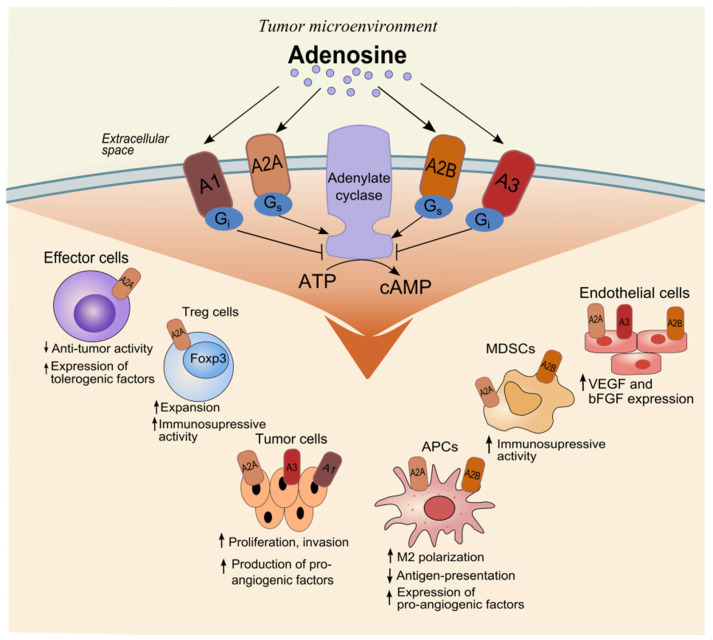
The effect of adenosine on tumor-infiltrating cells. Adenosine can bind to four different G-protein-coupled adenosine receptors that either stimulate (mediated by A2A and A2B adenosine receptors) or inhibit (mediated by A1 and A3 adenosine receptors) adenylate cyclase activity and cAMP production in the cell. Activation of adenosine receptors on various types of cells in the tumor microenvironment can lead to the formation of immunosuppressive conditions and inhibition of the anti-tumor immune response. Abbreviations: ATP: adenosine triphosphate; cAMP: cyclic adenosine monophosphate; Treg: regulatory T cells; Foxp3: forkhead box P3; APCs: antigen-presenting cells; MDSCs: myeloid-derived suppressor cells; VEGF: vascular endothelial growth factor; bFGF: basic fibroblast growth factor.

Tumor hypoxia and inflammation alter the microenvironment to regulate tumor growth and suppress antitumor immune reaction [19]. Adenosine can regulate both innate and adaptive immune responses [3]. Various transcriptional factors, signaling pathways, and cytokines contribute to the accumulation of adenosine in the TME [19,20,21,22]. Adenosine affects tumor cells and tumor-infiltrating cells by the activation of adenosine receptors (Figure 2).

Receptors A1 (A1R) and A2A are highly affine to adenosine. In physiological conditions, adenosine functions via these receptors, while A2B and A3 are low affine receptors [3]. The type of G proteins activated in the stimulated cells determines the effect of each adenosine receptor. A1R and A3R are associated with Gi, Gq, and Go proteins. The A1R or A3R ligation on the immune cells can activate immune reactions due to the inhibition of adenylate cyclase activity and a decrease in the levels of cAMP. A2AR and A2BR are Gs-coupled. They induce the enzymatic activity of adenylyl cyclase and increase the intracellular level of cAMP. Besides, through cAMP activation, adenosine receptors are associated with MAPK, PKA, and EPAC signaling that control gene transcription, metabolism, proliferation, and apoptosis, which are important for tumor progression [3,4].

Adenosine binds with A2AR at CD4^+^ and CD8^+^ effector T cells and natural killer (NK) cells, which suppresses their functions [3]. This limits their anti-tumor activity and induces the expression of immune regulatory molecules cytotoxic T-lymphocyte associated protein 4 (CTLA-4) and programmed cell death 1 (PD-1) [23]. It should be mentioned that in patients with cancer, stimulation of A2AR delays the maturation of NK cells and inhibits their cytotoxic activity [24,25]. The expression of Fas-ligands, granzyme b, perforin, and TNF-related apoptosis-inducing ligand on CD8^+^ T-cells decreases after binding of adenosine with A2AR [26]. The results of experimental studies demonstrate that the blockade of A2AR on T cells suppresses tumor progression [27,28].

On the contrary, A2AR and A2BR ligation on anti-inflammatory cells, such myeloid-derived suppressor cells (MDSCs) and regulatory T cells (Treg), induces their activation and expansion [29,30,31]. MDSCs and Treg cells can suppress the proliferation and functioning of effector cells and prevent the effective anti-tumor immune response. Besides, A2BR activation stimulates the polarization of macrophages into anti-inflammatory M2 type [32]. Such macrophages contribute to tumor progression and metastasis by producing immune-suppressing factors such as indoleamine 2,3-dioxygenase, arginase, and transforming growth factor β (TGF-β). Besides, A2BR and A1R activation on tumor-infiltrating neutrophils enhances the production of metalloproteases and contributes to metastasizing [32].

Some studies have highlighted the importance of the activation of adenosine receptors on tumor cells for their proliferation, survival, migration, and invasivity [33,34,35,36,37]. Besides, adenosine stimulates angiogenesis via A2BR and A2AR activation on endothelial cells [32] and angiotensin-mediated differentiation of dendritic cells (DC) [38,39]. For these reasons, adenosine receptors are actively studied. Currently, blockage of A2AR and/or A2BR, A3AR by monoclonal antibodies or small molecules is being studied in phase I-II of clinical studies on anti-tumor immunotherapy [4,37].

## 3. Adenosine Kinase

### 3.1. Biological Significance of ADK

ADK belongs to the family of ribokinases and is a key enzyme for the removal of extracellular adenosine by its phosphorylation into 5′-adenosine monophosphate (AMP) [15] (Figure 1). Regulating the availability of adenosine, ADK is an important link in complicated homeostatic and metabolic networks. The balance of adenosine and ADK is strictly maintained in healthy cells and the changes in the expression of ADK lead to the activation of adenosine receptors, which often determines the role of ADK in the development of pathologies [40].

Apart from its role in purine metabolism, ADK is involved in the regulation of transmethylation. During this biochemical reaction, the methyl group formed from the donor S-adenosylmethionine (SAM) is transferred onto an acceptor (lipids, dopamine, and DNA). The resulting product of all transmethylation reactions, S-adenosylhomocysteine (SAH), is further cleaved by SAH hydrolase (SAHH) into adenosine and homocysteine. In this reaction, adenosine is a by-product. If it is not completely deleted by ADK, it leads to the accumulation of SAH, which inhibits various transmethylation reactions necessary for a wide range of cellular processes [41]. The necessity of ADK enzyme for the organism was experimentally shown by Boison et al. [42]. ADK knockout mice had lethal phenotype. Homozygote ADK^−/−^ mice normally developed during embryogenesis. However, four days after birth, they developed microvesicular hepatic steatosis and die 14 days later, demonstrating a reduction in ATP deposits, inhibition of transmethylation reactions, and accumulation of adenosine. In humans, inborn deficiency of ADK is detected in patients with hypermethioninemia, encephalopathy, and pathological liver functioning, demonstrating disorders in methionine and adenosine metabolism [43,44]. On the contrary, overexpression of ADK and adenosine deficiency can lead to nervous system pathology, in particular, to the development of epilepsy because adenosine acts as an inhibitory neuromodulator for neuronal cells and has an anticonvulsant effect [45].

Being a regulator of transmethylation, ADK can participate in the modulation of DNA methylation. This epigenetic mechanism plays an important role in the embryonal and fetal development of mammals, and aberrant methylation of DNA is typical for some pathologies. In particular, its significance was shown in the cases of cancer and neurologic disorders [46]. Presently, the role of ADK in the methylation of DNA is not determined. According to the studies performed on experimental models, there is an association between the alterations in the expression of ADK and changes in DNA methylation in patients with epilepsy, brain lesion, vascular inflammation, angiogenesis, atherosclerosis, and cancer, as well as the influence of pharmacological blockade of ADK on the level of global hypermethylation [41].

ADK enzyme exists in two isoforms that are similar in biochemical and kinetic properties [47] (Table 1). Structurally, isoforms are identical excluding n-terminus, wherein the long isoform (ADK-L) contains additional 20–21 amine acids that are replaced by four amino acids in a short isoform (ADK-S). These isoforms are formed as a result of alternative splicing [48]. Western blot analysis of rat tissue showed that the expression of a long isoform dominated in the heart and spleen, and a short isoform in the brain. A high level of expression of both isoforms was established for the kidneys, liver, lungs, and pancreas [48].

Besides, ADK isoforms are characterized by various intracellular localization. ADK-L is located in the nucleus and ADK-S–in the cell cytoplasm [49]. Various localization of isoforms suggests differences in functioning. ADK-S controls the extracellular level of adenosine and mediates the degree of activation of adenosine receptors and ADK-L regulates epigenetic functions. ADK-L participates in the reaction of SAM-dependent transmethylation, which induces DNA and histone methylation. This is contributed by the presence of ADK-L, methyltransferase, and SAH in the cellular nucleus [41]. A high level and activity of ADK-L are associated with high global methylation of DNA [50]. It was shown that BHK-AK2 cells that express ADK-L demonstrate an increase in the global methylation of DNA by 400%, while the cells with ADK-S expression—only by 50%. These data suggest that both isoforms can be involved in epigenetic regulation.

Changes in the expression of isoforms of ADK play an important role in the development of the brain. During early postnatal development of the rat brain, the expression of ADK undergoes a coordinated revolutionary shift from the primarily neuronal to the primarily astrocytic pattern of expression. The studies showed a fast decrease in the expression of neuronal transcripts of ADK-L and the growth of astroglial transcripts of ADK-S within the first two weeks of postnatal development of the rat brain [51]. Besides, the main role in the expression of ADK-L was shown with an influence of an external irritant. The expression of an ADK-L isoform contributed to the regulation of proliferation of neuronal stem cells after traumatic brain damage [52].

Recently, the involvement of ADK in the biosynthesis of an important metabolic cofactor NAD^+^ had been demonstrated [53]. Dihydronicotinamide ribose (NRH) is a precursor for the synthesis of NAD^+^. It reduces into NAD^+^ via a pathway, wherein ADK acts as an NRH kinase. Pharmacologic or genetic inhibition of ADK blocks the synthesis of reduced nicotinamide mononucleotide and suppresses NRH-mediated biosynthesis of NAD^+^ in vitro and in vivo.

ADK, as an important regulator of adenosine level, modulated various cellular processes, including sleep homeostasis [54]. It was shown that mice with transgene overexpression of ADK spent less time sleeping in comparison with wild-type mice [55]. Another study on astrocyte-selective ADK knockout mice demonstrated an elevated level of homeostatic sleep drive associated with adenosine [56]. ADK acts as a highly sensitive and important metabolic sensor of glial ratio ATP/ADP and AMP, directly controlling the levels of intracellular adenosine.

In addition to nervous system disorders [50,52,57], the changes in the level of ADK enzyme attract researchers who study diabetes [58,59], vascular inflammation [60,61], and cancer [5,62].

### 3.2. The Role of ADK in Cancer

The survival and expansion of tumor cells have a close association with adenosine metabolism [5]. All adenosine receptors (A1, A2a, A2b, and A3) can take part in carcinogenesis, exerting a pro-tumor or anti-tumor effect. An increased level of adenosine in TME exerts an immune suppressing effect, primarily mediated via an excessive activation of the Gs-bonded A2A receptor on the immune cells [63]. Hypoxia-inducible factor 1α (HIF-1α) contributes to the accumulation of adenosine. Hypoxia–A2A-adenosinergic signaling inhibits the T-cell receptor–triggered production of proinflammatory cytokines (such as IFNγ) and redirects the immune response to the expression of immunosuppressive factors, in particular, a TGF-b, interleukin (IL)-10, CTLA-4, and activation of Treg cells [64]. ADK, as a regulator of the level of adenosine, can facilitate in carcinogenesis and affect immune processes (Table 2).

It was shown that in patients with cancer, the levels of adenosine in tumor cells are regulated primarily by ADK-mediated phosphorylation to AMP and not the effect of ADA. This process is hypoxia-independent [13]. However, currently, there is no clear understanding of the role of ADK in the development of tumors. Thus, it was shown that the tumor tissue of patients with colorectal cancer had an elevated level of ADK gene expression and ADK activity in comparison with healthy tissue [65,66]. Patients with glioma also showed enhanced expression of the gene and protein of ADK in both the nucleus and cytoplasm of tumor cells [67]. On the contrary, patients with liver cancer had decreased levels of ADK in tumor tissue in comparison with healthy tissue [68]. The expression of ADK isoforms was different. Besides, decreased expression of ADK was associated with liver cancer relapse. The study on a model of transgene mice with deficient expression of ADK in the liver revealed their enhanced sensitivity to acute toxic effects of a carcinogen (diethylnitrosamine) and associated lethality.

The differences in the expression of two ADK isoforms are observed in the cases of breast cancer [62]. The changes in the tumor tissue primarily concerned ADK-L in comparison with healthy tissue. Its expression was enhanced. Suppression of expression of ADK-L and ADK-S led to a decrease in cell proliferation, deletion, and migration of cultivated tumor cells of breast cancer. Knockdown of ADK-L gene decreases gene expression of matrix metalloproteases, cyclin D2, and molecules of adhesion, which suggests the potential role of ADK-L in the mitogenesis, cancerogenesis, and invasion. Besides, a recent study on the epigenetic role of ADK-L on tumor cell lines revealed a direct association between the expression of ADK and DNA methylation. The application of specific ADK inhibitors decreased the level of global DNA methylation in HeLa cells in a dose-dependent manner [69].

Another mechanism of ADK-mediated effect on tumors can be epigenetic regulation of pro-angiogenic factors. The formation of new blood vessels often occurs in response to hypoxia in the cases of cancer or ischemic disorders. Earlier, the study showed that hypoxia was associated with a suppression of ADK functioning and limitation of phosphorylation of intracellular adenosine to AMP [70]. Besides, deficiency of ADK in the endothelial cells increases the level of intracellular adenosine and stimulates DNA hypomethylation in the promoter regions of some pro-angiogenic genes, in particular, the gene of vascular-endothelial growth factor receptor 2 (VEGFR2). It leads to the proliferation and migration of cells in vitro, improves wound healing, and stimulates angiogenesis induced by ischemia in experimental animals in vivo [71]. Thus, the association between ADK and cell proliferation in the process of development, correlation of changes in the expression of this enzyme in the tumor tissue, as well as association of epigenetic changes that are observed during cancer, make ADK, in particular, ADK-L, a promising therapeutic target.

## 4. Adenosine Deaminase

### 4.1. The biological Significance of Adenosine Deaminase

ADA enzyme is an important protein in the purine metabolism and acts as a catalyzer and co-stimulator, as well as provides an intercellular connection [16]. ADA deaminates adenosine and 2′-deoxyadenosine to inosine and 2′-deoxyinosine, respectively. In humans, ADA has two isoforms, ADA1 and ADA2 (Table 1). ADA1 is found in all human tissues. It is highly expressed by T and B cells [72,73] and provides around 90% of all ADA activity. The main function of ADA1 is in the regulation of the intracellular level of adenosine. Mutations with the loss of function in the gene ADA1 can lead to hereditary immune deficiencies, known as severe combined immunodeficiency (SCID) in 15% of cases. SCID is associated with a dysfunction of circulating T, B, and NK cells and severe lymphopenia because of intracellular accumulation of toxic products of adenosine and cell apoptosis [73].

ADA1 can have an extracellular effect that binds to the surface of cells with dipeptidyl peptidase 4 (CD26) and adenosine receptors A1, A2A, and A2B on the surface of immune cells. This determines its enzyme-independent adaptive function. Due to a double bond with both CD26 on T-cells and with A2AR on DC, ADA1 can contribute to the formation of immunological sinapsis [74,75]. Moreover, its interaction with CD26 regulates lymphocyte-epithelial cell adhesion [76]. It is suggested that enzymatic activity of ADA1 is necessary for immune cells for the protection from suppression mediated by Treg cells that are involved in the generation of extracellular adenosine [77,78]. In lack of ADA1, adenosine can limit the proliferation of T cells and the secretion of cytokines by the activation of the A2AR. Besides, ADA1 can interact as an allosteric effector that controls the program of follicular T-helpers enhancing the affinity of adenosine to A1R (primarily expressed by T cells and B cells) and functionality of the receptor. This leads to controlled signaling to CD26/IL-2/IL-6 in addition to a decrease in the levels of adenosine and, thus, availability of metabolites for other adenosine receptors [79].

On the contrary, isoform ADA2 is not widely found in human organisms. Its structure, cellular localization, and expression are different from ADA1 [80]. It has a more open and hydrophilic catalytic center, which is reflected in enzymatic capacity. In particular, the ADA2 Km value is 100-fold higher for deamination of adenosine than ADA1. Besides, ADA2 is not more active than ADA1 at pH 6.9. Thus, in physiological conditions, its deamination function is limited. ADA2 works well in weak acidic acid, for example, in hypoxic conditions [80].

If ADA1 has intracellular localization, ADA2 forms homodimers and secrets into extracellular space. ADA2 is highly expressed in myeloid cells and produced by activated monocytes, macrophages, and DC in the sites of inflammation and TME [81]. Besides, ADA2 is secreted by monocytes during differentiation into macrophages or DC, and its secretion is regulated by interferon (IFN)-γ. Immune-inflammatory diseases such as systemic lupus erythematosis, rheumatoid arthritis, Chrone’s diseases, tuberculosis, and HIV infection, are associated with an enhancement in the activity of ADA2, which can be used as a biomarker of the disease and response to treatment, especially in patients with tuberculosis [82,83,84,85]. It is reported that ADA2 can act as a growth factor [86]. ADA2 exerts autocrine activity, induces the proliferation of monocytes, and contributes to the differentiation of anti-inflammatory macrophages M2 [87]. There is also indirect evidence of the possible role of ADA2 as an endothelial growth factor [88].

Mutations in the *ADA2* gene lead to an inflammatory disease called ADA2 deficiency (DADA2), which is characterized by inflammatory vasculopathy and early strokes that are frequently associated with hypogammaglobulinemia [73,89]. DADA2 is less expressed than SCID-ADA. DADA2 treatment includes hematopoietic stem cell transplantation. Without treatment, ADA1-deficient SCID is fatal at an early age. Its treatment primarily includes hematopoietic stem cell transplantation as well as enzyme replacement therapy and gene therapy [90]. Although the lack of functional ADA1 and ADA2 leads to a disturbance in the immune function regulation, the lack of one functional element is not compensated by the presence of the other. It suggests that ADA1 and ADA2 play different roles [73]. Kaljas et al. showed that this can be explained by binding with various immune cells [72]. ADA1 binds with T cells and NKT cells that express CD26 receptors, and ADA2 binds with neutrophils, monocytes, B cells, NK cells, and CD39^+^ Treg cells, that do not express CD26. Adenosine deaminases, which bind with the surface of these cells, can regulate their activity via a decrease in the concentration of extracellular adenosine. Besides, they can participate in the formation of immunological synapsis, regardless of their enzymatic activity.

Moreover, ADA plays a role in male fertility [91,92]. Another study that included newborn children showed that ADA1 and ADA2 demonstrated persistent changes within the first weeks and months of life and correlated with the levels of cytokines and chemokines in the plasma. Thus, ADA may play a functional role in the immune ontogenesis of a human [93,94]. ADA can be examined as a target in anti-tumor therapy due to immune-modulating properties and the ability to limit the concentration of adenosine.

### 4.2. The Role of ADA in Cancer

An intensification of ADA activity is observed in patients with different diseases, including cancer (Table 3). There is an association between a high activity of ADA and the stage of disease for gastric, bladder, breast, colorectal, and renal cell cancer [66,95,96,97,98,99,100,101,102]. It is reported that for some cancers, a decrease in the activity of ADA is associated with a progression of such diseases as head and neck, prostate, and laryngeal squamous cancer [103,104,105]. In the case of lung cancer, the significance of ADA activity is controversial. A decreased activity of ADA in the peripheral lymphocytes is observed in patients with stage IV cancer [106]. However, the level of ADA has a diagnostic significance in the bronchoalveolar lavage fluid and can be used as an additional parameter for the evaluation of malignancy when a biopsy can be complicated [107]. Noteworthy, the activity of ADA (ADA1 and ADA2) changes during the interaction of tumor cells (triple-negative breast cancer) with lymphocytes, macrophages, and endothelial cells in vitro, providing unfavorable phenotype and contributing to cancer progression. An association between the activity of ADA2 in the plasma of patients with breast cancer with a pro-tumor M2 phenotype of macrophages, as well as the activity of ADA1, and endothelial cell dysfunction or inflammatory parameters [102] was shown.

In patients with head and neck cancer, the possibility to limit the ADA activity and CD26 expression in effector T cells and CD3^+^ exosomes derived from T cells [105] has been seen. In the early stages of the disease, exosomes and T cells function relatively normally, but along with the progression of the disease, the expression of ADA/CD26 gets suppressed.

## 5. Targeting ADK and ADA in the Cancer Therapy

The accumulation of extracellular adenosine prevents effective anti-tumor immunotherapy [5]. Thus, enzymes that regulate the levels of adenosine can be useful tools for the development of new approaches that would improve the treatment effectiveness.

Enhancement of ADA1 expression can not only decrease the levels of adenosine in TME but also accumulate inosine. It was shown that T cells can use adenosine as an alternative source of carbon to synthesize glucose for the maintenance of their functions in glucose-deficient conditions in vitro [108]. The addition of inosine enhanced the anti-tumor effect of immune checkpoints (PD-L1/PD-1) and the adaptive transfer of T cells into tumors in the experimental models.

Recently, PEGylated ADA2 (PEGADA2) was studied on preclinical cancer models, providing promising results for clinical application [109]. In mice, PEGADA2 inhibited tumor growth depending on the level of the enzyme activity and affected the immune response. Another study showed that chimeric antigen receptor (CAR)-engineered T cells, which overexpress ADA, increased the resistance to exhaustion of CAR-T cells. At the same time, tumor burden decreased, which shifted TME to the direction of anti-inflammatory immunity in experimental models [110].

The application of ADA inhibitors for the treatment of malignant pathology has been reported. This approach can lead to the accumulation of adenosine in the sites of inflammation. Besides, it can reduce the production of free radicals, which are a known side-effect of numerous anti-tumor drugs. ADA can deaminate and inactivate adenosine analogs that are used in chemotherapy [16]. Thus, ADA inhibitors (pentostatin and cladribine) are applied in the treatment of a rare hematological malignant pathology—hairy cell leukemia [111]. Unlike pentostatin, another ADA inhibitor erythro-9-(2-hydroxy-3-nonyl) adenine (EHNA) can induce the apoptosis of malignant pleural mesothelioma (MPM), which is an aggressive tumor that does not have effective therapy. It was shown under experimental conditions that EHNA prevents the proliferation of MPM cells due to an increase in the level of extracellular adenosine in vitro and reduces tumor growth in mice inoculated with MPM cells in vivo [112]. The inhibition of ADA by 2′deoxycoformycin (dCF) has been reported to reduce tumor size and its growth in mice with modeled breast cancer in vivo and in human tumor cells in vitro [113]. dCF suppresses the migration and invasion of tumor cells via the activation of A2AR and A3R as well as the adhesion and transmigration of tumor cells through the layer of endothelial cells by the stimulation of A2AR.

Primarily, ADK is a therapeutic target for conditions such as epilepsy, pain syndrome, and inflammation. The protective effect of ADK inhibitors has been shown [114]. Lately, new data have appeared on the role of ADK-L in the regulation of cellular proliferation in the pancreas [58,115] and nervous system [52] as well the involvement of ADK-L in mitogenesis, carcinogenesis, and tumor invasion [62]. Besides, the epigenetic role of ADK-L has been shown and is actively studied. It is known that hypermethylation of DNA is a pathological feature of various cancers [116,117]. The inhibition of ADK-L can contribute to the reverse of DNA hypermethylation phenotype in cancer [69]. Modulation of ADK-S is also possible to overcome the immune-suppressive barrier in the TME because ADK-S can regulate the level of adenosine and activation of adenosine receptors [118]. Considering the changes that occur in the expression of ADK in the tumor tissue, it can be suggested that ADK is a potential diagnostic marker and a perspective target for anti-tumor therapy.

## 6. Conclusions

**Figure 3:**
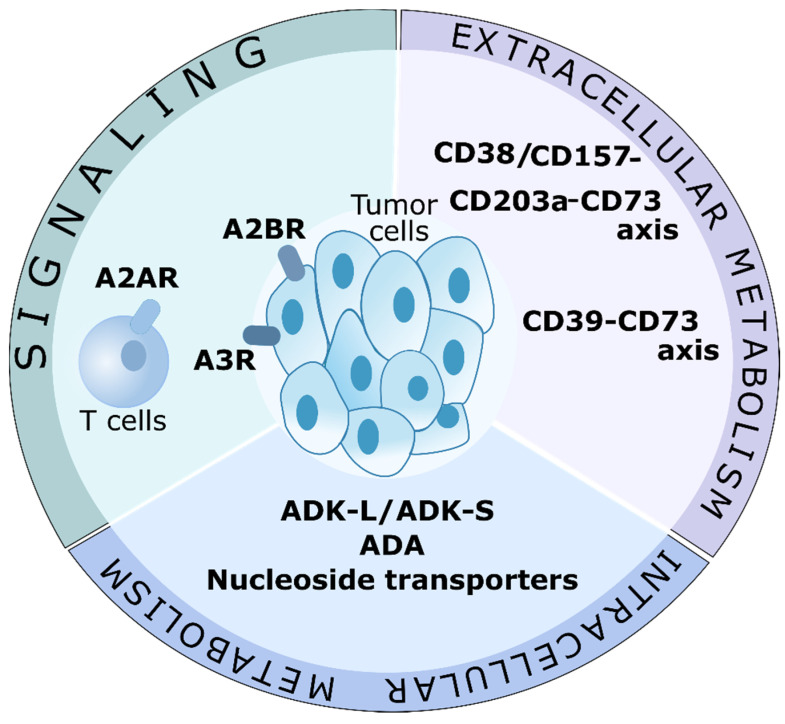
Targeting adenosine in cancer therapy. Possible adenosine-associated targets for cancer therapy. Signaling, and extracellular and intracellular metabolism may be involved in limiting the immunosuppressive action of adenosine. Possible therapeutic targets are A2A receptors (A2AR) on T cells, and A2B and A3 receptors on tumor cells. Like a part of the extracellular metabolism, the CD39-CD73 axis and the CD38/CD157-CD203a-CD73 axis are actively investigated in clinical trials. Targeting adenosine-cleaving enzymes, ADK and ADA, as well as nucleotide transporters, may become a new direction in cancer therapy. Of particular interest is ADK-L, which is involved in epigenetic regulation.

The regulation of the level of adenosine has therapeutic potential for cancer therapy (Figure 3). Currently, the studies experimentally and clinically confirmed the effectiveness of CD39-, CD73-blocking antibodies, and antagonists of adenosine A2A receptor as agents that decrease the levels of adenosine and block their signaling [5]. Inhibition of the adenosinergic pathway improves the effectiveness of blockade of other immune checkpoints, like PD-1/PDL-1 [119] or CTLA-4 [120] as well as the effectiveness of CAR-T cell therapy [121] and adaptive T cell therapy [122]. In experimental studies, combined therapy against CD73 and CD39 or CD73 and A2AR showed better response in comparison to monotherapy with CD73, CD39, or A2AR [123,124,125]. This indicates that one-target therapeutic agents do not prevent the complete generation of adenosine or signaling. It is generally accepted that tumor cells can form an immune-suppressing microenvironment with increased concentration of extracellular adenosine [4]. However, the intracellular metabolism of adenosine in patients with cancer is understudied. It is difficult to completely inhibit the synthesis of adenosine because of the variety of enzymes and metabolic pathways that contribute to its production. Targeting enzymes in the TME that decrease the levels of adenosine (such as ADA and ADK) can significantly prevent the activation of adenosine receptors and enhance cancer immunotherapy.

Considering its epigenetic role, ADK can become a promising target for the therapy of tumors with hypermethylated phenotypes. Since there are differences in the functioning of ADK-L and ADK-S, further studies are required on the role of each isoform in the maintenance of ATP/adenosine in cases with cancer. Currently, the main obstacle to such studies is a lack of isoform-specific inhibitors, in particular, highly selective for a nuclear isoform of ADK-L [41]. Besides, the contribution of ADK-S into DNA methylation is understudied [50].

Thus, the application of ADK and ADA can become a perspective approach to anti-tumor therapy. Currently, they are actively being studied for various malignant pathologies. It is necessary to have a clear and detailed understanding of the mechanism of action of these enzymes in tumor to introduce them in clinical practice.

## Figures and Tables

**Table 1:** Characteristics of ADK isoforms and ADA isoforms *.

	ADK		ADA	
	ADK-L (Isoform 1)	ADK-S (Isoform 2)	ADA1	ADA2
Gene (chromosome)	ADK (10q22.2)		ADA 20q13.12	ADA2 22q11.1
Protein structure	40.5 kDa monomer, 362-amino acid form	monomer, 345-amino acid form	40.8 kDa monomer, 363 amino acids	59 kDa monomer-homodimer, 511 amino acids
Tissue specificity	Widely expressed, occurs in large amounts in liver, heart, kidney, lung, pancreas, and spleen	Widely expressed, occurs in large amounts in liver, brain, kidney, lung, and pancreas	Found in all tissues, occurs in large amounts in lymphocytes and intestine	Human adult heart, lung, lymphoblasts, and placenta, fetal lung, liver, and kidney
Cell specificity	Ubiquitously, neuronal cells, glial cells		Ubiquitously, lymphocytes, erythrocytes	Myeloid cells
Cellular localization	Intracellular, nucleus	Intracellular, cytoplasm; plasma membrane	Intracellular; extracellular via CD26	Extracellular, secreted; lysosome
Functions	Catalyzes the phosphorylation of adenosine to AMP; Facilitates methylation reactions by the removal of adenosine, the end product of SAM-dependent transmethylation reactions	Catalyzes the phosphorylation of adenosine to AMP, using ATP as a phosphate donor and produces ADP and AMP; Acts as a regulator of concentrations of extracellular adenosine and intracellular adenine nucleotides	Catalyzes the hydrolysis of adenosine to inosine and 2-deoxyadenosine to 2-deoxyinosine; Acts as a positive regulator of T cell coactivation, by binding CD26; Enhances DC immunogenicity; Acts as a positive modulator of A1R and A2AR	May contribute to the degradation of extracellular adenosine Binds to cell surfaces via proteoglycans and may play a role in the regulation of cell proliferation and differentiation, independently of its enzyme activity
Km for adenosine	Approx. 1 µM	-	Approx. 37 µM	Approx. 2.25 mM
Disease at deficiency	Hypermethioninemia encephalopathy due to adenosine kinase deficiency		T-B-NK- severe combined immunodeficiency	ADA2 deficiency

* Based on the GeneCards database and The Human Protein Atlas.

**Table 2:** The role of ADK in carcinogenesis.

Cancer/Model	Parameter	Significance	Ref.
PC3 human prostate carcinoma cell line and MDA-MB-231 human breast adenocarcinoma cells	Protein level	The adenosine-ATP catalytic cascade is initiated via ADK-mediated phosphorylation of adenosine into AMP rather than its deamination to inosine	[13]
Breast cancer (*n* = 46 patients) and breast cancer MDA-MB-231 cell line	Protein level	ADK-L expression was significantly increased in breast cancer tissue; ADK downregulation suppressed proliferation, viability, migration, and invasion of cancer cells	[62]
Colorectal cancer (*n* = 10 patients)	Gene expression	ADK expression is higher in tumor than in healthy tissue	[65]
Colorectal cancer (*n* = 40 patients)	Enzyme activity	Higher in tumor than in healthy tissue (*p* < 0.01)	[66]
Glioma (*n* = 45 patients)	Gene and protein expression levels	In tumoral and peritumoral tissues, ADK expression was markedly elevated compared with that in control tissues (*p* < 0.05)	[67]
Liver cancer (*n* = 11 patients) and mouse model of hepatic ADK deficiency	Protein level	ADK in the liver might play a role in determining the liver’s susceptibility to cancer development	[68]
HeLa, HepG2, and U373 cancer cell lines	Protein level	HeLa cells combine the highest DNA methylation levels with the highest expression levels of ADK-L; ADK inhibitors significantly reduced global DNA methylation in HeLa cells	[69]

**Table 3:** The role of ADA in carcinogenesis.

Cancer/Model	Parameter	Significance	Ref.
Gastric cancer (*n* = 15 patients)	Enzyme activity	ADA activity increased in the cancerous tissues (*p* < 0.0005); there were no significant differences between I-II stages and III-IV stages	[95]
Gastric cancer (*n* = 26 patients)	Enzyme activity and protein level	ADA activity of the cancer gastric juices were lower (*p* < 0.01) and protein concentrations were higher than in the healthy control group	[96]
Bladder cancer (*n* = 40 patients)	Serum enzyme activity	ADA activity was significantly higher in cancer than in healthy controls	[97]
Bladder cancer (*n* = 36 patients)	Enzyme activity	Increased ADA activity was found in cancerous tissues compared with cancer-free adjacent tissues (*p* < 0.05)	[98]
Breast cancer (*n* = 160 patients)	Protein level	Level of serum ADA was higher compared with healthy control (*p* < 0.05); level of ADA was significantly reduced upon tamoxifen treatment (*p* < 0.05)	[99]
Breast cancer (*n* = 58 patients)	Enzyme activity	The mean values for ADA activity (tissue and serum) of patients with breast cancer were significantly higher than those of the benign breast disease (*p* < 0.005) and healthy subjects (*p* < 0.0001)	[100]
Breast cancer (*n*= 19 triple-negative breast cancer) and MDA-MB-231 triple negative breast cancer cells	Enzyme activity	Patients had higher plasma ADA2 activities and lower ADA1/ADA2 ratio at advanced stages of cancer development than in the initial stages; the activity of ADA changes during the interaction of tumor cells with lymphocytes, macrophages, and endothelial cells in vitro contributing to cancer progression.	[102]
Colorectal cancer (*n* = 40 patients)	Enzyme activity	Higher in tumor than in healthy tissue (*p* < 0.01)	[66]
Renal cell cancer (*n* = 33 patients)	Serum enzyme activity	ADA activity was significantly higher in patients than in the healthy group (*p* < 0.001)	[101]
Prostate cancer (*n* = 68 patients)	Serum enzyme activity	ADA activity in serum of patients with prostate cancer and patients with bone metastases were significantly decreased (*p* < 0.05) when compared with the healthy control group	[103]
Laryngeal cancer (*n* = 15 patients)	Enzyme activity	ADA activity was decreased in cancerous tissues when compared with the cancer-free adjacent tissues (*p* < 0.025)	[104]
Head and neck squamous cell carcinomas (*n* = 14)	Protein level	With progression of the disease, the expression of ADA/CD26 in effector T cells and CD3^+^ exosomes derived from T cells gets suppressed	[105]
Lung cancer (*n* = 13 patients with advanced stage)	Enzyme activity	Patients with advanced stage of lung cancer exhibited a decrease in ADA activity in both lymphocyte and erythrocyte (*p* < 0.005)	[106]
Lung cancer (*n* = 43 patients)	Enzyme activity	ADA levels in bronchoalveolar lavage fluids were statistically higher compared with the non-malignant group (*p* < 0.001) and may be a diagnostic biomarker in lung malignancies	[107]

## Data Availability

Not applicable.
